# Controversies in the Management of Endometrial Cancer

**DOI:** 10.1155/2010/638165

**Published:** 2010-06-16

**Authors:** V. Masciullo, G. Amadio, D. Lo Russo, I. Raimondo, A. Giordano, G. Scambia

**Affiliations:** ^1^Division of Gynecologic Oncology, Catholic University of Sacred Heart, 00168 Rome, Italy; ^2^Center for Biotechnology, College of Science and Technology, Temple University, Philadelphia, PA 19122, USA

## Abstract

Endometrial cancer (EC) remains the most common malignancy of the female genital tract. The median age at diagnosis is the sixth decade, with abnormal uterine bleeding at the presentation in 90% of the patients. Surgical treatment, including complete hysterectomy, removal of remaining adnexal structures, and an appropriate surgical staging, represents the milestone of curative therapy for patients with EC. Adjuvant therapy is necessary in patients at high risk of recurrence. Conservative treatment approaches should be used in selected cases for women with a desire of fertility preservation. This review summarizes the management of EC and discusses current controversies regarding the role of lymphadenectomy and radiotherapy in patients with intermediate-risk tumors confined to the uterus.

## 1. Introduction

**Figure 1:**
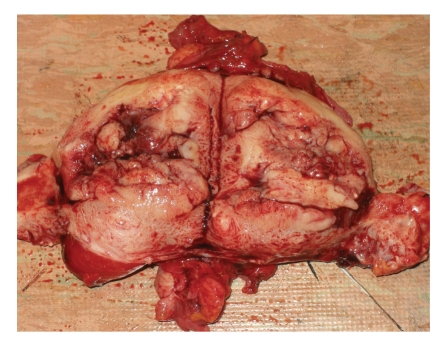
Adenocarcinoma of the uterine corpus.

Endometrial cancer (EC) remains the most common malignancy of the female genital tract. It will develop in 2,6% of women in the United States during their lifetime [1]. The age-standardized death rate is 3.6 per 100,000 women and the median age at diagnosis is the sixth decade, although 20 to 25% of cases will be diagnosed premenopausally [2, 3] (Figure 1). It has been suggested that the overall distribution of tumour stage and survival are similar for younger and older women; however, women with stage I disease and younger than 45 years are more likely to have low-grade disease localized to the endometrium [4, 5].

## 2. Risk Factors

The most important risk factors for EC are postmenopausal status, excessive fat consumption, body mass index (BMI) of 25 kg/m^2^ or more, nulliparity, anovulation, and unopposed exogenous estrogen use. However, only half of patients present with identifiable risk factors, while the other half appear to be at low risk [6].

In particular, obesity, recently considered the most common risk factor responsible of the development of all endometrial carcinomas, increases the risk of EC through a number of mechanisms that cause hormonal alteration and consequently endometrial cell proliferation, apoptosis inhibition, and angiogenesis promotion. In premenopausal women, obesity causes insulin resistance, ovarian androgen excess, anovulation, and chronic progesterone deficiency. On the other hand, in postmenopausal women, the conversion of androgens to estrogens is enhanced in peripheral fat stores. Pregnancy, with intense placental production of progestins and grand multiparity protect against EC, whereas nulliparity increases the risk, especially when it is associated with infertility. It is well established that oral contraceptives with the addition of progesterone to estrogen, lower the adverse effects of estrogens on the endometrium and the risk of EC [7]. Smoking appears to reduce the risk of EC through its effects on estrogen production and metabolism [8]. Instead, the use of tamoxifen in patients with breast cancer triples the risk of EC and also increases the chance of developing benign endometrial polyps, hyperplasia, and even carcinoma in some patients. However, the beneficial effects of tamoxifen on breast cancer recurrence and its association primarily with ECs of low grade and early stage support its continued use in an appropriate patient population [1].

While the incidence and mortality rates from several other cancers have plateaued or decreased in the last decade, rates for EC continue to rise [9]. This fact may be related to an increased rate of advanced-stage cancers and high-risk histologies including uterine papillary serous carcinoma (UPSC), that is histologically similar to serous epithelial ovarian carcinoma and represents approximately 10% of all endometrial cancer [10].

Bokhman [11] proposed the existence of two categories of endometrial carcinoma characterized by distinct microscopic appearance, epidemiology, and clinical behaviour. Type I carcinoma with an endometrioid histology, that typically arises in relatively younger women with obesity, hyperlipidemia, and signs of hyperestrogenism (endogenous or exogenous); and type II carcinomas that include poorly differentiated endometrioid, clear cell, and serous histologies. They often arise in thinner, older women and demonstrate no hormonal risk factors. Moreover type I endometrial carcinomas are commonly diagnosed at an early stage and have a favourable prognosis, often with surgical treatment alone; recurrences are usually local (pelvis being the most common site) and frequently curable with tumor-directed radiotherapy. Alternatively, type II endometrial carcinomas are more likely to present with metastatic disease at diagnosis and carry a poorer prognosis [12].

## 3. Diagnostic Approach

Abnormal uterine bleeding is present in 90% of patients with EC. Therefore, any vaginal bleeding in a postmenopausal woman warrants an initial evaluation for EC, that is found approximately in 10% of patients with postmenopausal bleeding (PMB) [1]. Because of this symptom, 75% of ECs are diagnosed at an early stage. Atypical endometrial hyperplasia (AEH) is felt to be a precursor of lesion and it may progress, over time, to EC in 5% to 25% of patients. In addition, AEH is associated with a coexisting EC in approximately 20% of patients [13, 14].

Diagnostic approaches to the assessment of abnormal uterine bleeding are divided into invasive and noninvasive methods.

### 3.1. Invasive Methods

#### 3.1.1. Dilatation and Curettage (D&C)

Traditionally considered the standard for investigation of abnormal uterine bleeding.

#### 3.1.2. Endometrial Biopsy

A variety of instruments (the Pipelle, the Pipette, the Tis-U-Trap and the Z-sampler) has been developed over the last decade for using in the office as alternatives to the expense, risk, and inconvenience of fractional D&C. With the use of these devices, the sensitivity for detecting endometrial cancer ranges from 67% to 96%.

#### 3.1.3. Hysteroscopy and Directed Biopsy

Some consider this method as the standard for the diagnosis of abnormal uterine bleeding. However, a recent study of 373 patients which retrospectively compared hysteroscopy and D&C, concluded that hysteroscopy did not improve upon the sensitivity of D&C in the detection of endometrial hyperplasia or carcinoma. On the contrary, Clark et al. found that hysteroscopy is highly accurate and useful in diagnosing, rather than excluding, endometrial cancer in women with abnormal uterine bleeding [15]. A recent study performed by Bedner and Rzepka-Gorska compared the effectiveness of D&C with hysteroscopy and guided biopsy in perimenopausal women at risk of endometrial hyperplasia or cancer. They found that hysteroscopy with directed biopsy was more sensitive than D&C for detecting all types of uterine lesions [16]. Several retrospective studies have found increased positive peritoneal cytology in women who underwent hysteroscopy, but recent studies have indicated that there is currently no evidence to suggest that diagnostic hysteroscopy increases the risk of malignant cells spreading into the peritoneal cavity, or worsens the prognosis in women with EC.

### 3.2. Noninvasive Methods

#### 3.2.1. Ultrasonography

Two large studies of 930 women and of 138 women reported experiences with transvaginal ultrasound in women with postmenopausal bleeding. Both studies used a biendometrial (double layer) thickness of four millimetres as a cut-off point. The sensitivity was 96% to 98% and the specificity was 36% to 68%. The false positive rate was 44% to 56%. Thickness could not be measured in 3% to 4.7% but the reason for this was not stated. One of the studies reported two cancers in patients with a thickness less than 3.5 mm, giving a false negative rate of two per 930 (0.2%) [2].

#### 3.2.2. Endometrial Cytology

This is not felt to be useful in diagnosis of EC, due to low accuracy and it will not be discussed further. In a prospective study conducted by Karlsson et al. [17], on 1168 women with PMB underwent to transvaginal ultrasonography followed by uterine curettage, the risk of endometrial abnormality was 6.1%, considering a threshold of 5 mm or less, with a sensitivity of 94%, a specificity of 78%, a positive predictive value (PPV) of 69%, a negative predictive value (NPV) of 96%, and a rate of accuracy of 84%. With this threshold, it was determined a risk of endometrial abnormalities of 6,1% (upper 95% confidence level of 8.5%) and ECs were undetected. The high NPV of this test lends itself well to excluding a diagnosis of EC in patients who cannot undergo endometrial sampling. However, it should be emphasized that the aforementioned results are limited to patients with PMB. Screening for EC using transvaginal ultrasonography alone in asymptomatic postmenopausal women has a poor PPV (9%) and is not recommended, whereas the combination of transvaginal ultrasonography and endometrial biopsy has shown a sensitivity of 100% [18].

Evaluation for systemic disease is typically limited to chest radiography and laboratory evaluation performed in preparation for surgery, but magnetic resonance imaging (MRI), that is superior to computed tomography for visualizing uterus and pelvic tissues [19], is recommended as knowledge of extrauterine spread or cervix involvement by tumor. On the other hand, baseline cancer antigen levels can be useful, but they are not enough sensitive to predict the status of disease. We recently showed [20] that transvaginal sonography (TVS) when carried out by expert hands shows a comparable accuracy to MRI in depicting myometrial infiltration of endometrial carcinoma, thus we recommend a combination of both techniques for detecting an accurate myometrial invasion. In detection of subclinical nodal disease, to define extent of disease, integrated PET/CT imaging has been investigated by Montejo et al. and only modest improvement was achieved over to conventional imaging, with an overall sensitivity and specificity of 50% and 86.7%, respectively [21].

## 4. Treatment of Precursor Lesions

Continuous stimulation of the endometrium by either endogenous or exogenous estrogen is the most important risk factor for endometrial hyperplasia and EC consequently. The World Health Organization (WHO) classifies the endometrial hyperplasia in simple, a benign proliferation of endometrial glands involving mild or moderate glandular crowding (adenomatous hyperplasia) and complex, that is characterized by back-to-back cellular crowding and an irregular cellular outline. Both simple or complex hyperplasia could be associated with cellular atypia. It can be subdivided into mild atypia (nuclear enlargement and rounding with evenly dispersed chromatin) or moderate atypia (larger nuclear size, prominent nucleoli, and clumped chromatin). Hyperplasia without atypia, either simple or complex, has a low likelihood (1% and 3%, respectively) of progressing to carcinoma. In contrast, atypical endometrial hyperplasia is believed to be the direct precursor to endometrioid EC [11, 12].

A recent investigation by The Gynecologic Oncology Group (GOG) [22] found that from 19% to 62% of endometrial biopsy specimens interpreted as atypical endometrial hyperplasia were associated with an invasive EC at hysterectomy. For this reason, simple and complex hyperplasia can be treated with progestational therapy only, whereas hysterectomy is mandatory for all patients with atypical hyperplasia. Medroxyprogesterone acetate or megestrol acetate, the agents used in most retrospective studies to treat endometrial hyperplasia without atypia, can be administered in either a cyclic or continuous fashion.

Atypical hyperplasia regresses after treatment with progestins in 60% to 95% of patients [23]. However, because of the high rate of frankly invasive EC in patients with atypical hyperplasia [24] and the high risk of progression to EC, hysterectomy is the standard treatment, while progestins therapy should be reserved for those women who desire a fertility-preserving management. Continuous administration of local progestational agents via the levonorgestrel (LNG)-releasing intrauterine device (IUD) has been evaluated as an alternative delivery mechanism in treating endometrial hyperplasia, both with and without atypia. It has an efficacy of 100% with lasting results during a minimum of 5 years of follow-up, although only small numbers of patients were included in the studies published to date [25].

The LNG-releasing IUD has also been evaluated as an alternative to hysterectomy for women with low-grade, presumed early-stage EC who are poor operative candidates. Cure rates up to 75% have been reported [26]. The current committee opinion from the American College of Obstetrics and Gynecology acknowledges that larger studies are needed to evaluate the efficacy of noncontraceptive uses of LNG-releasing IUDs before they can be recommended as a treatment alternative for atypical endometrial hyperplasia or low-grade EC. Follow-up endometrial biopsy or curettage is performed every 3 to 6 months until regression to normal endometrium or lesion progression occurs [27]. However, well-designed randomized trials for an optimal endometrial hyperplasia management are lacking, and guidelines for follow-up are also unclear. If vaginal bleeding resumes, another endometrial biopsy should be performed [28, 29].

## 5. Fertility-Sparing Treatment of Endometrial Cancer

Considering that patients with stage I disease and younger than 45 years are more likely to have low-grade disease localized to the endometrium [30], a conservative management of uterine cancer has been advocated as a safe alternative for those women with desire of childbearing. Anyway, there is still no consensus about which will be the optimal procedure.

We recently proposed an innovative method [31] to preserve fertility in young women with stage IA EC, based on the hysteroscopic resection of the tumor followed by hormone therapy regimen of megestrol acetate (160 mg/day) for six months, for consolidation. This methods consists of a conservative resectoscopic treatment using a three-step technique in which each step is characterized by a pathologic analysis: (1) the removal of the tumor, (2) the removal of the endometrium adjacent to the tumor, and (3) the removal of the myometrium underlying the tumor. This technique, under a close postsurgical follow-up, might represent a novel therapeutic option. The results of transvaginal ultrasound examination and diagnostic hysteroscopy with target biopsies at 3, 6, 9, and 12 months after surgery were negative for atypia or malignancy and four out of six patients (66%) achieved childbearing. 

Moreover successful hormone therapy as an option for appropriately selected young women who desire to preserve fertility, with early-stage low-grade endometrial cancer, has been reported in small series [32, 33].

This conservative management of EC should not be considered standard of care, and the dosage and duration of treatment, selection criteria, and follow-up surveillance are not established definitely. In a 2004 meta-analysis, Ramirez et al. reviewed the literature regarding hormonal treatment of grade I EC, including 27 articles with a combined total of 81 patients. A variety of progestational agents were used, most often medroxyprogesterone acetate or megestrol acetate. It was observed an overall response rate of 77% (62/81), the median time to regression was 12 weeks and among responders the recurrence rate was 24% [34].

All recurrences occurred within 1 year of diagnosis and all patients who remained disease free (76% of the initial responders) required treatment with progesterone for only 1 month to achieve a response. Twenty patients achieved pregnancy after treatment. The 23% (19/81) of patients of the original cohort never responded to treatment, and only 68% had any documented follow-up endometrial sampling. Today there is no clear consensus on the optimal follow-up interval. However, appropriate patient selection and exclusion criteria remain undefined, so patients must be counseled that failure to identify recurrence or extension of disease during progestational treatment could lead to a delay in definitive surgery and ultimately a compromised prognosis [35].

On the other hand, progestational therapy can be used successfully to treat patients with atypical hyperplasia and well-differentiated presumed stage I EC while preserving fertility.

## 6. Surgical Treatment of Endometrial Cancer

Surgical treatment, including complete hysterectomy, removal of remaining adnexal structures, and appropriate surgical staging represents the milestone of curative therapy for patients with EC. Survival is heavily dependent on surgical stage, which is determined adopting the classification recently revised by the International Federation of Gynecology and Obstetrics (FIGO) in 2008 (Table 1).

Most women with endometrial cancer have disease confined to the uterus and they are usually managed with extra-fascial or simple total hysterectomy with bilateral salpingo-oophorectomy (BSO) either as a laparotomic or laparoscopic procedure. Lymph node involvement is an adverse prognostic factor; it is influenced chiefly by the depth of myometrial invasion and the tumor grade. Regarding the role of lymphadenectomy in women with disease that clinically seems to be confined to the uterus, there has been much debate. Although lymphadenectomy forms part of the International Federation of Gynecology and Obstetrics (FIGO) surgical staging system [28], evidence from a large randomized controlled trial, A Study in the Treatment of Endometrial Cancer (ASTEC), showed that this approach does not provide therapeutic benefit [29].

Panici et al. conducted a randomized clinical trial to determine whether the addition of pelvic systemic lymphadenectomy to standard hysterectomy with bilateral salpingo-oophorectomy improves overall and disease-free survival. They found that significantly it improved only surgical staging and neither overall or disease-free survival [36].

A comparison of other two important studies, GOG-99 and Postoperative Radiation Therapy in EC (PORTEC), seems to suggest that lymphadenectomy does not affect disease-related and recurrence-free survival in patients with intermediate-risk tumors confined to the uterus [7, 37]. However, 60% of the patients enrolled in the PORTEC trial were actually grade 1 (thus their prognosis was even more favourable) whereas doubts have been raised concerning the adequacy of surgical staging performed in the GOG-99 trial.

When the EC presents a cervical extension (stage II FIGO), a radical hysterectomy, with an extensive dissection to expose the ureters and secure the uterine vessels at the origin rather than at their entry into the uterus, may be considered. This type of surgery allows to remove the parametrial tissue and facilitates safe dissection of the bladder away from the uterus and cervix such that a significant cuff of upper vagina can be removed [38]. Parametrial metastasis does not form part of the FIGO staging system, but their involvement is associated with a poor prognosis [28].

Mariani et al. reported the results of 34 women with surgical stage II EC treated primarily with surgery. The disease-free survival at 5 years was 100% for women who had radical hysterectomy with histologically negative nodes versus a 5-year disease-free survival rate of 73% for women who received simple hysterectomy [39].

Boente also evaluated 202 patients with stage II disease, reporting a 5-year survival for radical hysterectomy and nodal dissection of 86% compared with 77% for simple hysterectomy [40].

However, some questions about the adequacy of lymphadenectomy, like the minimum number of nodes to remove, if the para-aortic nodes should be resected and if the hystotype of endometrial cancer should determine the extent of lymphadenectomy, remain still unclear.

A small number of women are found to have advanced endometrial cancer at presentation thus, to date there are no prospective randomized data available to aid general consensus about an appropriate management of these patients. The appropriate extent of surgery in this setting of patients and the true value of radical surgery in advanced disease are still not clear. Pliskow et al. published a retrospective study of 41 women with clinical stage III and IV endometrial cancer, and suggested that the extent of disease and tumour bulk have greater prognostic value than histological subtype, grading, or depth of myometrial invasion. Other recent studies propose that the primary cytoreductive surgery for advanced endometrial cancer offers a survival benefit as in epithelial ovarian cancer [41, 42].

## 7. Radiotherapy

The role of adjuvant radiotherapy in EC remains controversial. Early endometrial cancer with low-risk pathological features can be successfully treated by surgery alone. Several trials, which have mainly included women at intermediate or high risk of recurrence in stage 1, have been shown that postoperative radiotherapy is able to reduce the risk of isolated local recurrence without improving recurrence-free or overall survival. In particular the PORTEC and the GOG-99 trial randomized patients with intermediate risk stage 1 showing that external pelvic radiotherapy (EBRT) improves local control but does not substantially increase survival in patients with EC confined to the uterus, with or without surgical staging [37, 43]. 

The ASTEC trial, randomizing patients with IC-IIA or IA-IIAG3 or serous papillary/clear cell for lymphadenectomy, did not show a survival benefit for adjuvant radiotherapy in women with intermediate- or high-risk early stage EC. Thus, the use of postoperative radiotherapy should be limited to patients with sufficiently high-risk of local recurrence based on known risk factors such as age ≥ 60, grade 2-3, depth of myometrial invasion, and cervical and lymphovascular space involvement. The PORTEC-2 trial, which compared the efficacy of brachytherapy (BRT) versus EBRT in patients with intermediate- or high-risk early stage EC, concluded that BRT is effective as EBRT in preventive vaginal recurrences with less toxicity. Therefore BRT should be considered the standard for these patients. However, as in the original PORTEC trial, surgical staging is not required, raising questions on the generalizability of these data. In fact, if an appropriate surgical staging is not performed, the administration of pelvic RT could lead to overtreatment of those patients who have negative lymph nodes and undertreatment of those with positive pelvic lymphnodes who eventually present disease in the para-aortic area.

Patients with advanced EC (stage IIB, III FIGO) should be considered for adjuvant external beam radiotherapy that would reduce local recurrence, with or without vaginal vault brachytherapy [44].

Bruckman et al. reported a retrospective review on EC patients treated with adjuvant pelvic RT and low-dose rate vaginal brachytherapy. Patients with extrauterine disease limited to the ovary or fallopian tube had significantly improved relapse-free survival and overall survival compared with those patients with disease spread beyond the adnexa to other pelvic structures. Women with extrauterine disease limited to the adnexa experienced relapse-free survival rates of 80% and overall survival rates of 80% compared with 15% and 40%, respectively, for those patients with disease to other pelvic structures beyond the adnexa [45].

## 8. Chemotherapy in Postoperative Treatment of Endometrial Cancer

The use of chemotherapy for patients with locally advanced or metastatic EC is becoming nowadays more common. Platinum compounds, taxanes, and anthracyclines provide the major effective drug classes in the treatment of advanced and recurrent EC, all producing response rates of 20% to 30%. For patients able to tolerate aggressive therapy, multiagent chemotherapy produces higher response rates than single-agent therapy [46]. The most active regimen tested in randomized trials is the triplet consisting of cisplatin (50 mg/m^2^), doxorubicin (45 mg/m^2^), and paclitaxel (160 mg/m^2^), a myelotoxic regimen, which requires granulocyte growth factor support [47].

Carboplatin and paclitaxel are used frequently because of their ease administration and promising phase 2 results. The GOG is currently comparing cisplatin-doxorubicin-paclitaxel chemotherapy to carboplatin-paclitaxel in a large randomized trial of patients with metastatic disease (GOG-209).

In a prospective study by the GOG in patients with relapsed or metastatic EC, doxorubicin and cisplatin were chosen to compare chemotherapy to radiotherapy [48]. All women with stage III or IV disease of any histology and with less than 2 cm of residual disease after maximal surgical debulking were eligible for the trial. Patients were randomized to whole abdominal radiotherapy or 8 cycles of doxorubicin and cisplatin chemotherapy. Toxicity was higher in the chemotherapy group; only 63% of women completed all 8 cycles of chemotherapy. Patterns of failure analysis revealed that the initial site of failure was within the pelvis in 13% of patients who underwent irradiation versus 18% of those who received chemotherapy. To further improve the results of chemotherapy alone, the GOG-184 study, required all patients to receive tumor-directed radiotherapy (pelvic irradiation with or without para-aortic irradiation, depending on lymph node involvement) followed by randomization to cisplatin and doxorubicin or cisplatin, doxorubicin, and paclitaxel. This trial has been completed and data are awaiting maturation. It is hoped that the combination of chemotherapy and targeted radiotherapy will improve on historical results.

An important trial coordinated by the European Organization for Research and Treatment of Cancer (EORTC) was recently presented in abstract form at the annual meeting of the American Society of Clinical Oncology [49]. Women with stage I to IIIA or IIIC (pelvic lymph nodes only) disease who were at high risk (>50% myometrial invasion, Grade 3 or DNA nonploidy, clear or serous histology) were randomized to external pelvic radiotherapy or combined chemoradiotherapy. During a 10-year period, 372 patients were enrolled, with a median follow-up of 3.5 years. The investigation was closed early because of slow recruitment, and multiple chemotherapeutic regimens were allowed in combination with radiotherapy. The hazard ratio for progression-free survival was 0.58 for chemoradiation (95% confidence interval [CI], 0.34–0.99; *P* = .046). This translates to an estimated absolute difference in 5-year progression-free survival of 7% (from 75% [95% CI, 67%–82%] to 82% [95% CI, 73%–88%]). The ongoing PORTEC-3 trial is also investigating whether chemoradiation is superior to radiation alone [50]. Patients are randomized to receive pelvic radiotherapy or pelvic radiotherapy with concurrent cisplatin followed by adjuvant carboplatin and paclitaxel chemotherapy. Because many patients with recurrent or stage IV EC are elderly, have received prior pelvic radiotherapy, or have limited hematologic reserve, chemotherapeutic regimens are often limited by toxicity.

The possible role of adjuvant hormonal therapy for stage I EC has also been investigated but currently the evidences are still insufficient [51] to reach any conclusion.

## Figures and Tables

**Table 1:** Carcinoma of the corpus uteri (FIGO 2008).

Stage I*	Tumour confined to the corpus uteri.
IA*	No or less than half myometrial invasion.
IB*	More than half myometrial invasion.
Stage II*	Tumour invades cervical stroma, but does not extend beyond the uterus.**
Stage III*	Local and/or regional spread of the tumour.
IIIA*	Tumor invades the serosa of the corpus uteri and/or adnexae^#^.
IIIB*	Vaginal and/or parametrial involvement^#^.
IIIC*	Metastases to pelvic and/or para-aortic lymph nodes^#^.
IIIC1*	(i) Positive pelvic nodes
IIIC2*	(ii) Positive paraortic lymphnodes with or without positive pelvic lymphnodes.
Stage IV*	Tumor invades bladder and/or bowel mucosa, and/or distant metastases.
IVA*	Tumor invasion of bladder and/or bowel mucosa.
IVB*	Distant metastases, including intra-abdominal metastases and/or inguinal lymph nodes.

*Either G1, G2, or G3.

**Endocervical glandular involvement only should be considered as Stage I and no more as Stage II.

^#^Positive cytology has to be reported separately without changing the stage.
